# Supplementary figures and images for: Spatial and longitudinal tracking of enhancer-AAV vectors that target transgene expression to injured mouse myocardium
Source: bioRxiv. 2025 Jul 9:2025.04.28.651096. Preprint. [Version 2] doi: 10.1101/2025.04.28.651096 (PMC12247933; doi:10.1101/2025.04.28.651096)

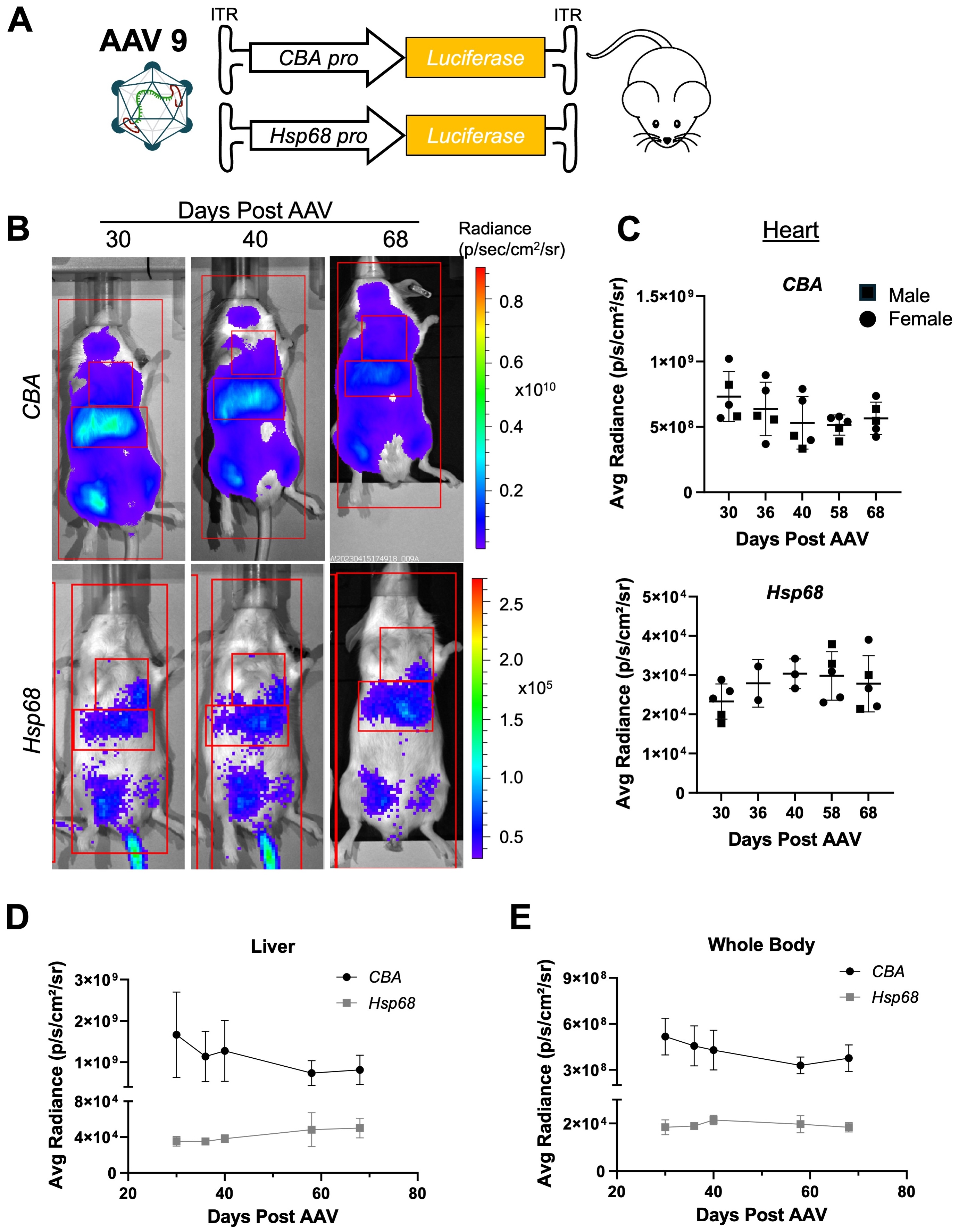

Supplement: Supplement 1 — Figure S1. IVIS imaging for tracking spatiotemporal expression of rAAV vectors (A) Schematic showing comparison of AAV9 vectors packaging either the strong, constitutively active chicken beta actin (CBA) promoter or minimal heat shock protein 68 (Hsp68) promoter to direct fLuc expression. n = 5 mice/AAV group. (B) Representative IVIS images of mice injected with AAV containing either CBA (top) or Hsp68 (bottom) promoters. Red boxes indicate ROIs marking cardiac, liver, and whole-body expression. (C) Average radiance measured from cardiac ROIs from CBA (top) or Hsp68 (bottom) promoters show relatively consistent expression from 30–68 days post-AAV injection. Square, male mice. Circle, female mice. (D, E) Average radiance measured from liver (D) and whole body (E) ROIs showed relatively consistent levels of expression over time for both promoters. [file media-1.jpg]

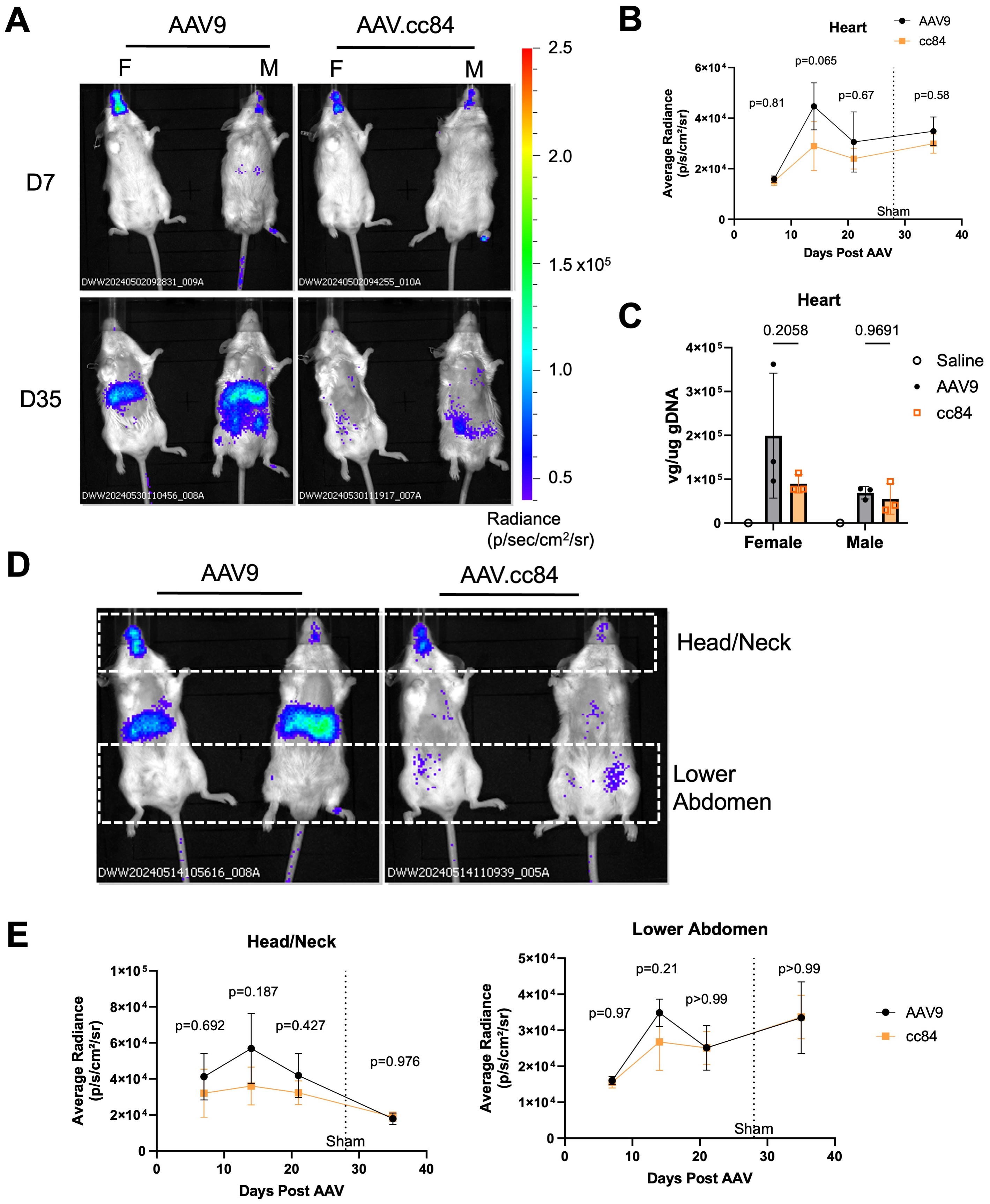

Supplement: Supplement 2 — Figure S2. AAV.cc84 capsid retains cardiac tropism while minimizing liver transduction (A) Representative IVIS images of mice injected with either AAV9 (left) or AAV.cc84 (right) at 7 (top) and 35 days (bottom) post-AAV injection. (B) Average radiance in the heart was similar between AAV9 and AAV.cc84 (n = 6 mice, Holm-Sidak multiple comparisons test). (C) Vector genome quantification from cardiac tissues showed similar levels of vector genomes between AAV9 and AAV.cc84 for both sexes (n = 3 mice, Holm-Sidak multiple comparisons test). (D) Representative IVIS images of mice with ROIs used to measure expression in head/neck and lower abdomen (white dashed box). (E) Average radiance measured in the head/neck (left) and lower abdomen (right) was similar between AAV9 and AAV.cc84 over the course of the study (n = 6 mice, Holm-Sidak multiple comparisons test). [file media-2.jpg]

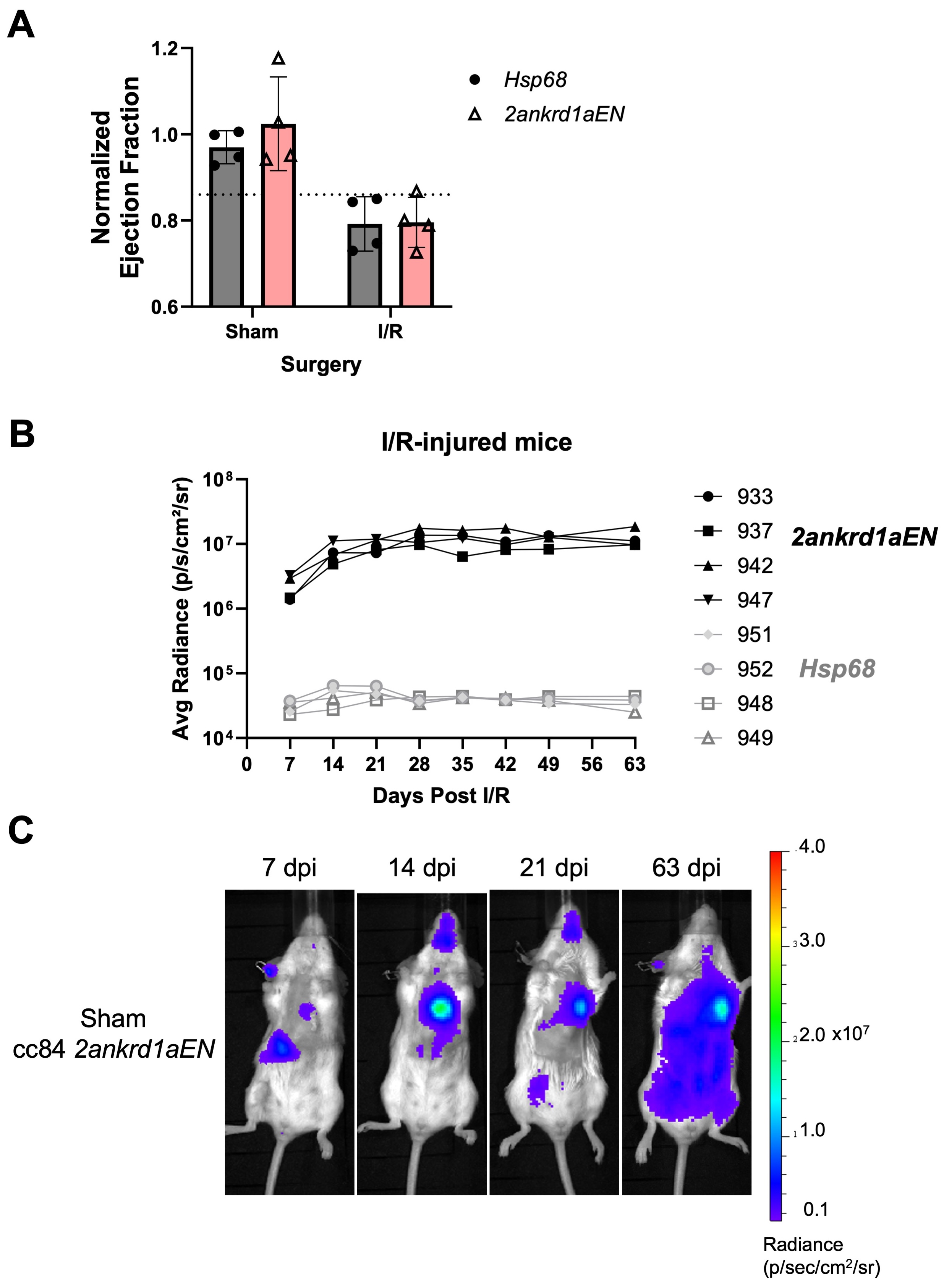

Supplement: Supplement 3 — Figure S3. Delivery of AAV.cc84 packaging 2ankrd1aEN after myocardial injury (A) I/R surgery injury extent was assessed by ejection fraction via echocardiography to estimate injury prior to AAV delivery (n = 4 mice). (B) Cardiac average radiance plotted for each individual mouse with I/R injury plotted over time with either Hsp68::fLuc (gray) or 2ankrd1aEN-Hsp68::fLuc (black). (C) Representative IVIS images of sham-operated mice injected with AAV.cc84 packaged with 2ankrd1aEN-Hsp68::fLuc. [file media-3.jpg]

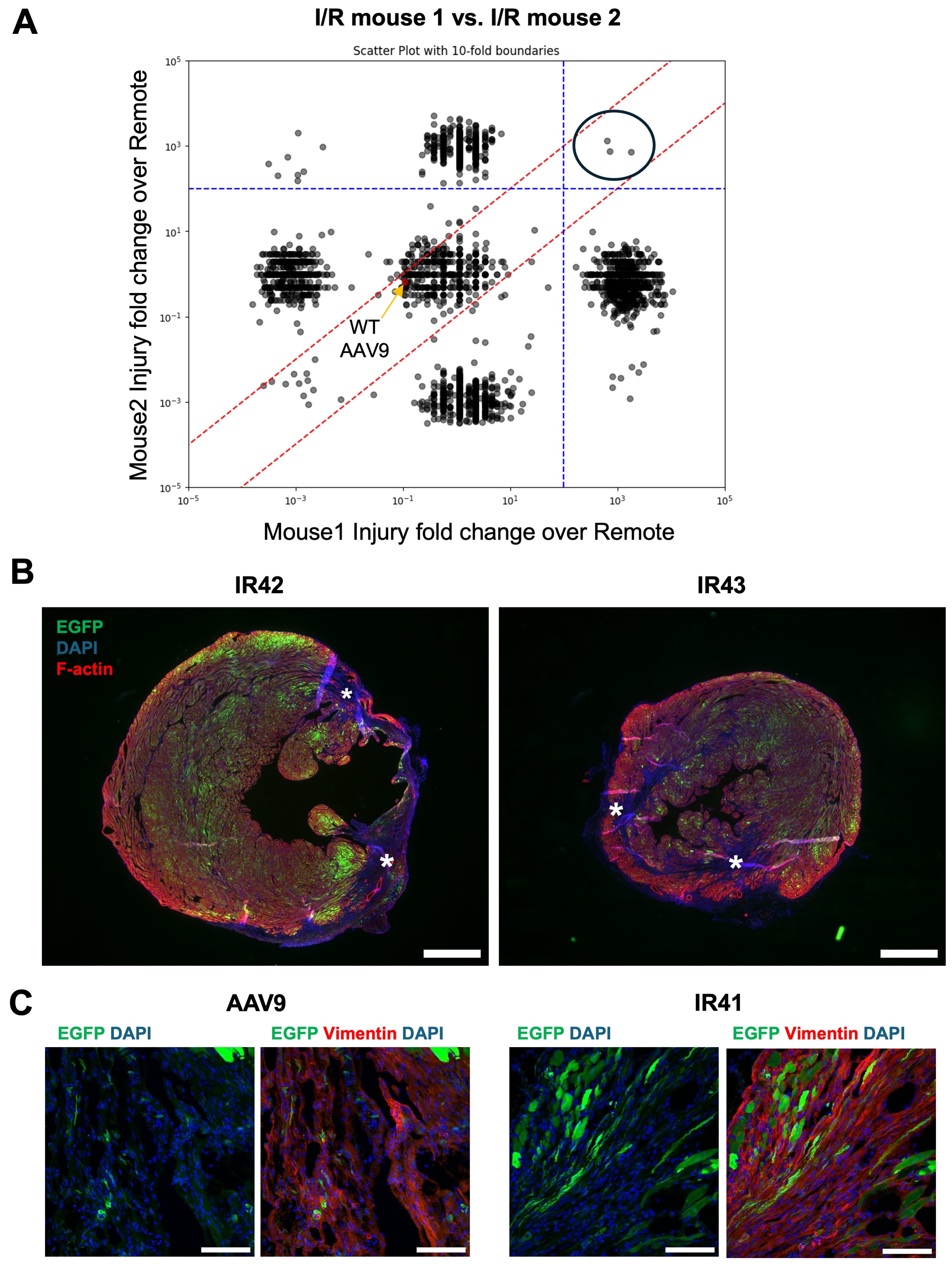

Supplement: Supplement 4 — Figure S4. Screening AAV capsid libraries enriched in injured myocardium (A) Injury site-enriched capsids in I/R-injured mice. Each point represents a unique capsid. Top left quadrant, capsids co-enriched at injury site. Wild-type AAV9 capsid is marked by an orange arrow. (B) Representative fluorescence imaging of variant capsids, IR42 (left) and IR43 (right), delivering a self-complementary CBA::EGFP cassette at 16 dpi. Asterisks indicate infarction area. Scale bar, 1 mm. (C) Immunofluorescence of infarct sites of mice transduced with either AAV9 (left) or IR41 (right) show no colocalization of EGFP and Vimentin. Scale bar, 100 um. [file media-4.jpg]

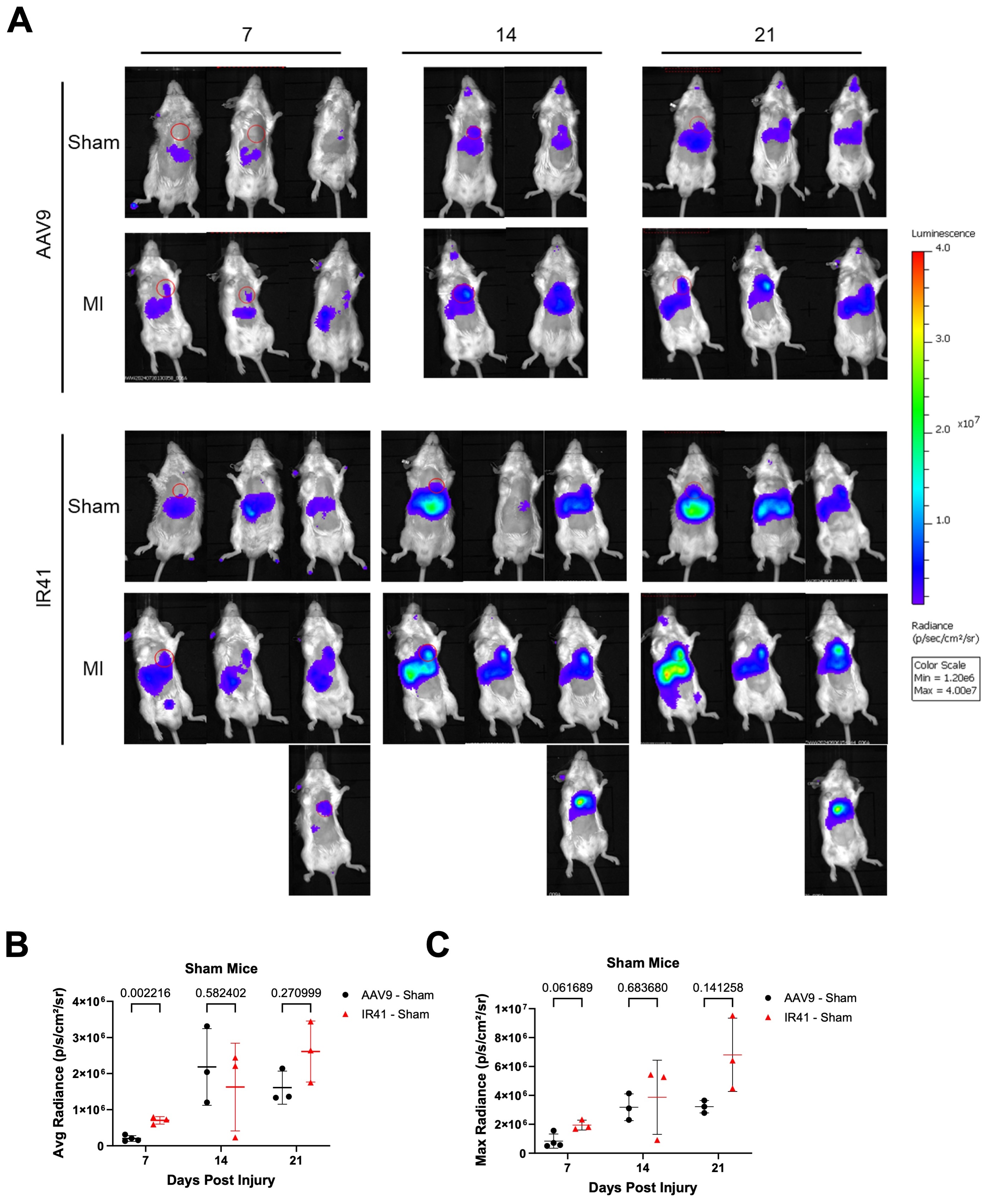

Supplement: Supplement 5 — Figure S5. Post-injury delivery of variantAAV.IR41 variant capsid enhances 2ankrd1aEN-directed expression in injured myocardium over AAV9 (A) Compiled IVIS images of mice that underwent sham or MI surgery with AAV9 or IR41 transduced at 3 dpi. Mice were imaged at 7, 14, and 21 dpi. (B, C) Cardiac average (B) and maximum (C) radiance were statistically similar between AAV9 and IR41 in sham-operated mice. [file media-5.jpg]
